# Entomopathogenic fungus disrupts the phloem-probing behavior of *Diaphorina citri* and may be an important biological control tool in citrus

**DOI:** 10.1038/s41598-022-11789-2

**Published:** 2022-05-13

**Authors:** Nathalie Maluta, Thiago Castro, João Roberto Spotti Lopes

**Affiliations:** 1 Department of Entomology and Acarology, Luiz de Queiroz College of Agriculture, University of São Paulo, C.P. 9, Piracicaba, SP 13418-900 Brazil; 2 Koppert Biological Systems, Rodovia Margarida da Graça Martins s/n-Km 17,5, Piracicaba, SP 13400-970 Brazil

**Keywords:** Entomology, Animal behaviour

## Abstract

Citrus is among the most important fruit crops worldwide; however, numerous pests and diseases affect the orchards, increasing production costs. The psyllid *Diaphorina citri*, is a vector of the phloem-limited bacteria ‘*Candidatus* Liberibacter spp.’, the causal agent of Huanglongbing (HLB) disease. The lack of a cure for HLB requires management of the vector, mainly by intensive use of chemical insecticides, leading to the selection of resistant populations. Our study determined the effects of the entomopathogenic fungus *Cordyceps fumosorosea* on the probing behavior of *D. citri* at different time points after the fungus was applied by spraying. The electrical penetration graph technique was used to monitor the stylet activities of *D. citri* after application of the microbiological product. The effects were more pronounced between 30 and 96 h after the insects were sprayed, with significant disruption of the stylet activities related to the phloem and directly associated with the transmission of HLB. Our study indicated that the microbiological product Challenger^®^, with the active ingredient *C. fumosorosea* fungus, can significantly change the probing behavior of *D. citri*, may be helpful in more-sustainable management of the vector, and can be used to reduce the spread of HLB.

## Introduction

Citrus is one of the most important fruit crops in the world economically important for Brazil, which ranks among the main citrus producers after China, and is the largest producer of fresh oranges and orange juice^1^. However, citriculture is affected by many phytosanitary problems that cause significant losses and increases in production costs.

A serious pest in citriculture is the Asian citrus psyllid (ACP), *Diaphorina citri* Kuwayama (Hemiptera: Psyllidae), which causes little direct damage but is the main insect vector in citrus orchard, transmitting the phloem-limited α-proteobacteria '*Candidatus* Liberibacter asiaticus' and '*Ca*. L. americanus’, causal agents of the disease known as Huanglongbing (HLB) or citrus greening^2,3^. HLB is highly destructive and affects practically all existing commercial citrus cultivars, causing significant economic losses^3,4^. In the Americas, this disease was detected for the first time in 2004 in the State of São Paulo, Brazil^5,6^ and in 2005 in the State of Florida, USA^7^.

*‘Candidatus* Liberibacter spp.’ can be disseminated through grafting/vegetative propagation of infected plants and by the ACP, and the severity incidence of HLB is dependent on *D.citri* densities and movement from infected to non-infected citrus trees. These phytobacteria have a close relationship with their vector species and are transmitted in a persistent-propagative manner to new plants through insect feeding, being acquired when the insect ingests phloem sap of the infected citrus tree and inoculates the bacteria into healthy plants via its saliva^8–10^. The HLB-associated bacteria can be acquired by the vector after a few minutes of feeding on infected plants; the longer the feeding period, the greater the transmission efficiency^11^.

The Foundation of Citrus Growers of São Paulo State (FUNDECITRUS) estimated that the average incidence of symptomatic orange trees for HLB in 2019 was 19%, rising to 20.9% in 2020 in the citrus belt of São Paulo and southwestern Minas Gerais, Brazil^12^. Because no curative methods to HLB are available, the disease is managed by planting healthy citrus seedlings, using HLB-resistant rootstocks^13^, eliminating infected plants to reduce the source of inoculum, and controlling the insect vector *D. citri* with chemical insecticides. This last is the main management method used by citrus producers^14,15^.

Indiscriminate use of chemical insecticides to control *D. citri*, in addition to being economically unsustainable for producers, often generates imbalances in the agroecosystem, interfering with the management and biological control of other citrus pests. A wide variety of natural enemies could be used in the biological control of this psyllid species, thus reducing the need for massive applications of traditional insecticides. Among the main biological-control agents we can mention: parasitoid species such as *Tamarixia radiata* (Waterston, 1922) (Hymenoptera: Eulophidae) and *Diaphorencytus aligarhensis* (Shafee; Alan; Argawal, 1975) (Hymenoptera: Encyrtidae)^16,17^; predatory insects, mainly of the orders Coleoptera, Neuroptera and Diptera^18–20^; and entomopathogenic microorganisms^21–23^.

Among microorganisms, entomopathogenic fungi are the main microbiological agent of natural mortality of *D. citri* and spread horizontally among insects, being able to infect different pest species. *Cordyceps fumosorosea* (Wize) Kepler, Shrestha & Spatafora (Hypocreales: Cordycipitaceae) (basionym: *Isaria fumosorosea)*, is a generalist species of fungus, commercially available and with high potential for *D. citri* control^24,25^.

Several studies have reported high insect mortality after *C. fumosorosea* application^25–29^. Others have evaluated the effects of traditional insecticides on vector probing behavior, with implications for the transmission of phytopathogens^10,30–32^. However, very few have investigated the effects of entomopathogenic fungi on the probing behavior of insect vectors before they are killed by the fungi^33–35^. These studies provide essential information for managing phytopathogens and their vectors since disruptions in stylet activities can affect the efficiency of transmission of pathogens to new host plants via vector feeding.

The probing behavior of sap-sucking insects can be monitored using the electrical penetration graph (EPG) technique^36,37^, which shows the stylet penetration into plant tissues in real-time on a computer monitor. The graph (waveforms) is later analyzed and correlated with biological activities, including the transmission of phytopathogens^10,31,34^.

In the present study, we hypothesized that by coming into direct contact with the insect (before its death), the entomopathogenic fungus *C. fumosorosea* would cause physiological and behavioral changes in the ACP *D. citri* due to the infection process, modulating the insect's stylet activities, affecting the ability to feed normally on phloem, and possibly reducing the chances of HLB transmission to new plants by bacteriliferous psyllids. We investigated if the fungus could modulate the stylet and probing activities of the ACP at different time points after spraying, allowing us to detect possible effects of the fungus on the insect´s probing behavior and the main period when the fungus affects the probing behavior before the insects die. The results would indicate if this biological-control agent would be efficient only in reducing psyllid populations or could also be an important means of reducing ACP feeding activities and/or HLB transmission in citrus orchards.

## Material and methods

### Biological material: *D. citri* colony and plants

A colony of healthy *D. citri* was maintained in screened cages with an aluminum frame (35 × 35 × 53 cm) and acrylic door, containing orange jasmine plants, *Murraya paniculata* (L.) Jack (Rutaceae) in a climate-controlled room (25 ± 2 °C; photoperiod of 14L:10D; relative humidity 60–70%), under fluorescent lights at Escola Superior de Agricultura “Luiz de Queiroz” (ESALQ-USP), Piracicaba, Brazil. Healthy ‘Rangpur lime’ seedlings were used for the feeding-behavior assays. Each insect and seedling were used only once in the EPG assays.

### Biological insecticide application

Before the probing behavior assays, adults (1–7 days old) of *D. citri* from the maintenance colony were placed in a glass tube and immobilized for 2–4 min in crushed ice. Thirty psyllids (not sexed) were placed in the center of an open Petri dish lined with filter paper. The insects were sprayed with a solution of the microbiological insecticide Challenger^®^ (*Cordyceps fumosorosea*, strain ESALQ 1296) (Koppert Biological Systems) at the dose recommended by the manufacturers (400 µl of the commercial product/100 ml of water). Approximately 1.5 ml of solution (water + commercial product) was sprayed on each group of psyllids (at 1 m between the insects and the spray bottle). To ensure fungus infection, after the EPG recording the insects were placed in a humid chamber to certify sporulation, and only insects with confirmed mortality caused by *C. fumosorosea* were used in the analyses.

As a control, the same protocol was used but the insects were sprayed only with water. The time points used were: 0, 15, 30, 48, 72, 96, and 120 h after insect spraying, conforming to the general biological phases of the fungus (adhesion, germination, penetration, vegetative growth)^38–40^. Each time point was composed of the treatment with *C. fumosorosea* (treated insects) and its control (untreated insects). The insects were sprayed in groups of 30 and then kept in cages containing Rangpur lime seedlings to fulfill the necessary period for the EPG experiment, except for time point 0-h, when the insects were used immediately after spraying. Treated and untreated psyllids were kept in separate cages and rooms (25 ± 2 °C; photoperiod of 14L:10D) to avoid contamination.

### Stylet activities and probing behavior of *Diaphorina citri*

The probing behavior of *D. citri* was evaluated in real-time using the EPG technique to monitor stylet activities at different time points after *C. fumosorosea* application.

To prepare the insects for the EPG assay, healthy adult females of *D. citri* (1–10 days old) from a colony maintained on orange jasmine were enclosed in a glass tube placed in an ice bath for 2–4 min to facilitate manipulation and then immobilized in a vacuum chamber under a dissecting microscope. Then, a gold wire (3 cm long, 18 µm in diameter; EPG Systems, Wageningen, The Netherlands) was attached to the psyllid pronotum with a small droplet of water-based silver glue. The opposite end of the gold wire was glued to a thin copper wire (2 cm long), which was connected to the EPG probe. Another copper electrode (10 cm long, 2 mm wide) was inserted into the soil of the plant.

After a 1-h starvation period, each psyllid was placed individually on the abaxial surface of a young Rangpur lime leaf. The EPG waveforms were recorded for 8 h inside a Faraday cage (for electrical noise isolation) in a climate-controlled room (25 ± 1 °C) using a Direct Current eight-channel EPG device, model Giga-8d, with Stylet+ for Windows software (EPG Systems). Twenty-two replicates were analyzed per each treatment (treated and untreated insects) at each time point. Also, the mortality of the insects analyzed in the feeding behavior assays were evaluated after 24 h of the beginning of each EPG recording.

### Statistical analysis

The EPG data were analyzed according to the waveforms described for *D. citri* by Bonani et al.^41^: non-probing (waveform np); intercellular apoplastic stylet pathway and salivary sheath secretion (waveform C); first phloem contact (waveform D); salivation into the phloem (waveform E1); passive phloem-sap ingestion (waveform E2); and active intake of xylem sap (waveform G).

The output of EPG recordings given by the EPG-Excel Data Workbook 5.0 of Sarria et al.^42^ for each insect was used to calculate the treatment mean for the sequential and non-sequential variables of each EPG. The selected EPG variables (mean ± SE) were calculated and compared between treatments as described by Backus et al.^43^: PPW: proportion of individuals that generated a particular waveform type; NWEI: number of waveform events per insect; WDI: total waveform duration (min) per insect; and sequential variables: ‘Time to 1st probe from the start of EPG’, ‘Number of probes to the 1st E1’, and ‘Time from start of EPG to 1st E’.

Before analysis, normality and homogeneity of variance were checked, and when necessary, the data were transformed with ln (x + 1) or √ (x + 1) to reduce heteroscedasticity and improve normal distribution. All parameters were analyzed with a parametric Student’s t-test (data with Gaussian distribution) or a nonparametric Mann–Whitney U test (data with non-Gaussian distribution). A chi-squared test was used to analyze the proportion of individuals that generated a particular waveform type (PPW). All data were analyzed using IBM Statistics SPSS 22.0 software^44^.

The insects treated with *C. fumosorosea* vs. untreated insects (control) were compared at the same time point. A combined analysis was not performed because the experiments at each time point were performed separately; in addition, due to the post-spray period, the individuals did not have the same age at the different time points.

The EPG records considered valid for statistical analysis were those in which: (a) the insects were on the plant and glued to the wire after 8 h of recording; (b) the insects performed some feeding activity in at least 5 of the 8 h evaluated; (c) insects that, after recording, were placed in a humid chamber and sporulated (confirming infection by *C. fumosorosea* or were negative for the untreated insects, ensuring that the changes in behavior were due to infection by the fungus.

### Ethical approval

The authors have permission to use all biological materials used in this study. Also, all methods were carried out in accordance with relevant guidelines and regulations.

## Results

### 0 h after spraying

**Figure 1:**
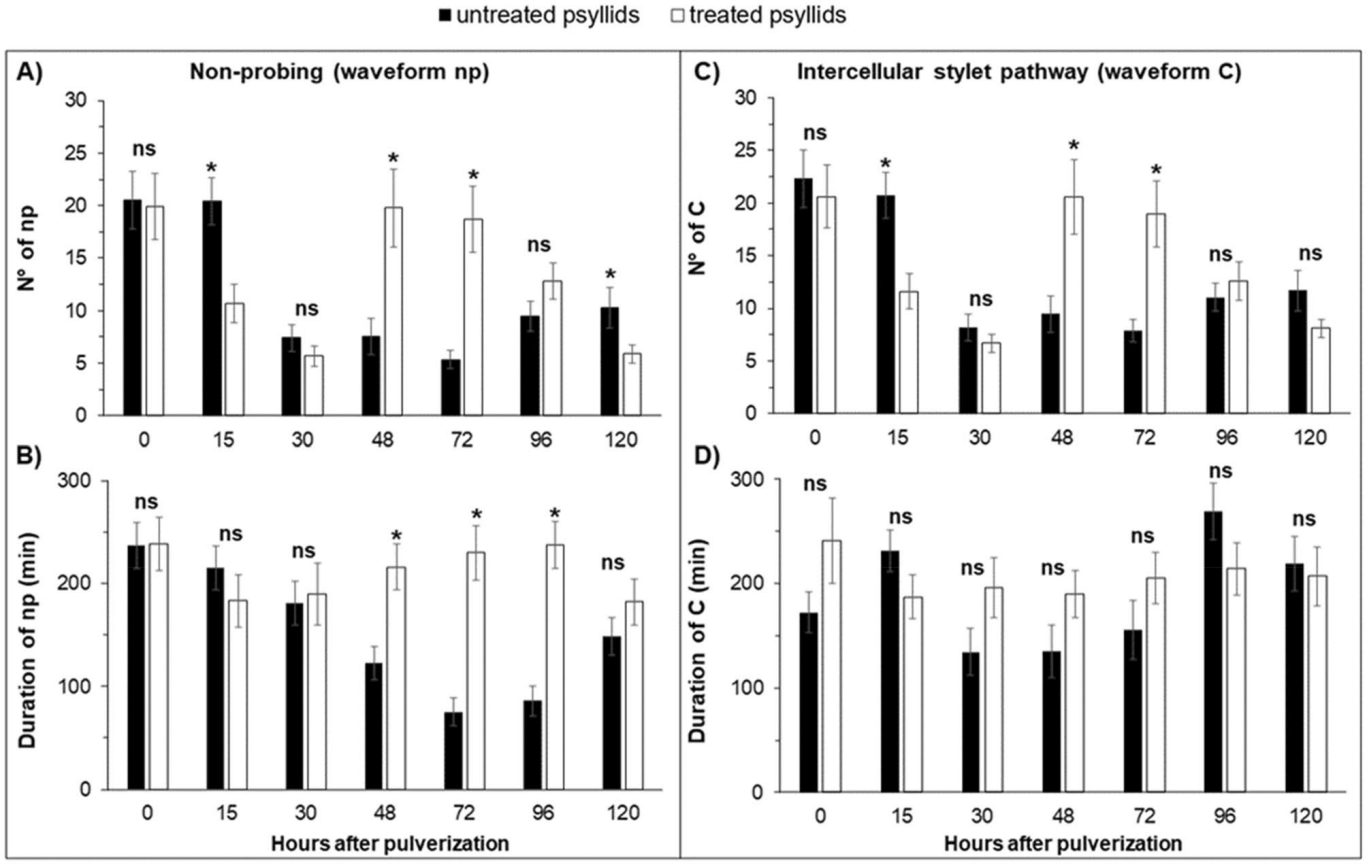
Number of waveform event per insect (NWEI) and Total waveform duration per insect (WDI) of non-probing (**A**,**B**) and waveform C (intercellular stylet pathway) (**C**,**D**) of Asian-citrus-psyllids *Diaphorina citri* on ‘Rangpur lime’ seedlings at different time points after microbiological insecticide pulverization (*Cordyceps fumosorosea*, strain ESALQ 1296). The columns and bars represent the mean and the standard error for each variable and time point. Bars with an asterisk (*) indicate a statistically significant difference (P < 0.05) according to t-Student test or Mann–Whitney U test between untreated vs. treated psyllids in the same time point (*ns* non-significant).

**Figure 2:**
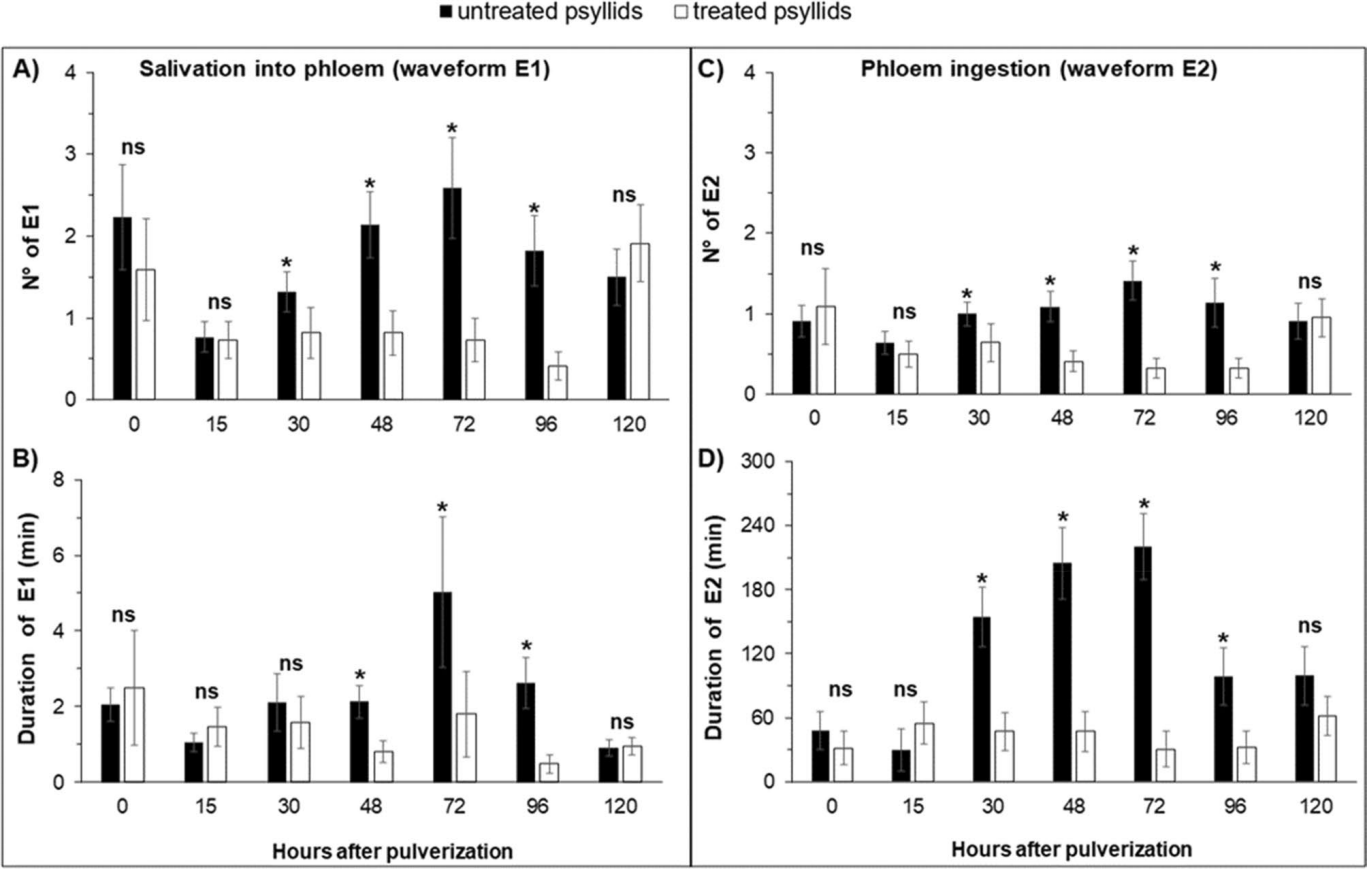
Number of waveform event per insect (NWEI) and Total waveform duration per insect (WDI) of E1 (phloem salivation) (**A**,**B**) and E2 (phloem ingestion) (**C**,**D**) of Asian-citrus-psyllids *Diaphorina citri* on ‘Rangpur lime’ seedlings at different time points after microbiological insecticide pulverization (*Cordyceps fumosorosea*, strain ESALQ 1296). The columns and bars represent the mean and the standard error for each variable and time point. Bars with an asterisk (*) indicate a statistically significant difference (P < 0.05) according to Mann–Whitney U test between untreated vs. treated psyllids in the same time point. (*ns* non-significant).

In the experiment with ACP at the time point 0-h after spraying, that is, sprayed and immediately connected to the EPG system to record the activities, no differences were observed in non-sequential variables, for number (NWEI) and total duration (WDI) of non-phloem (wave C, np, G) (Fig. 1) and phloem activities (D, E1 and E2) (P > 0.05) (Fig. 2; Supplementary material Table S1) and in the proportion of insects (PPW) that performed salivation into the phloem (E1) (x^2^ = 1.504; P = 0.36) and ingested phloem sieve (E2) (x^2^ = 1.467; P = 0.36) (Fig. 4).

**Figure 3:**
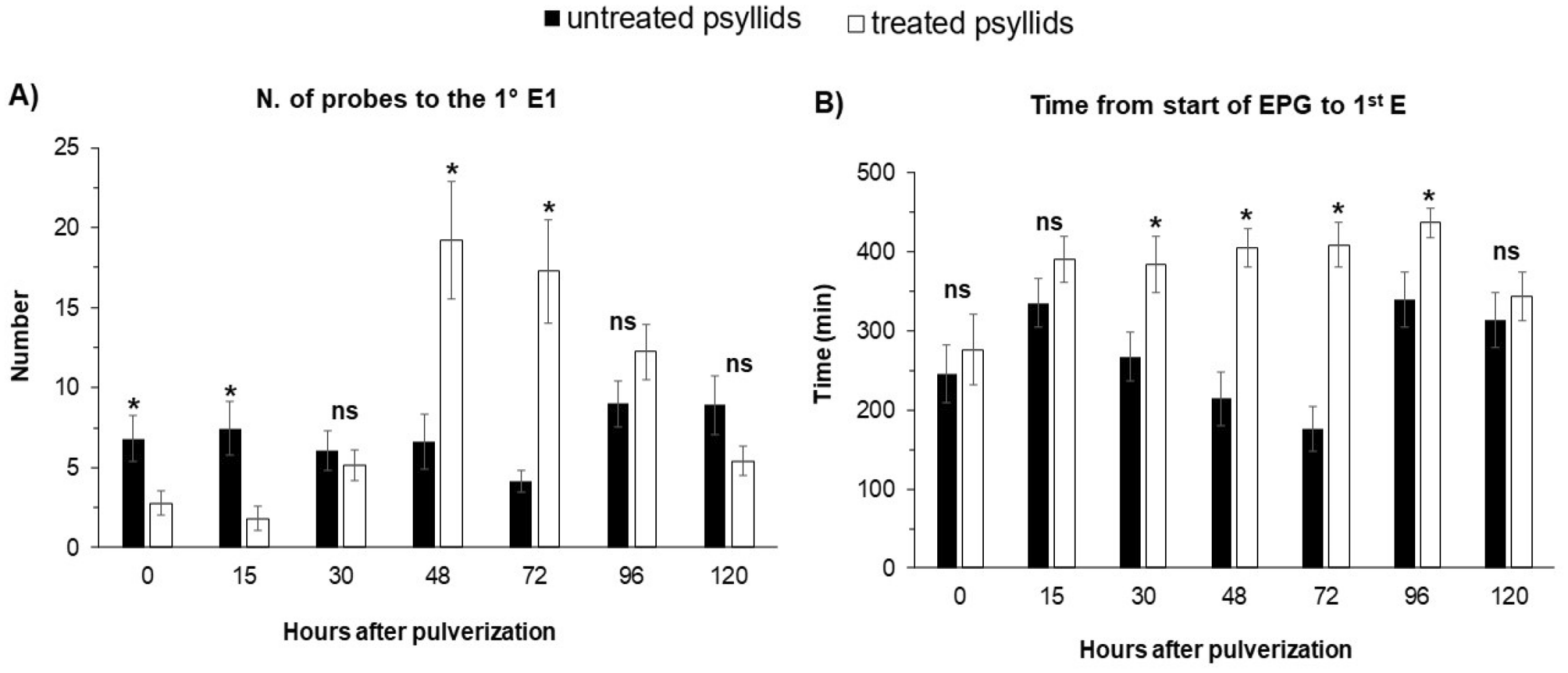
EPG sequential variables of probing behavior of Asian-citrus-psyllids *Diaphorina citri* on ‘Rangpur lime’ seedlings at different time points after microbiological insecticide pulverization (*Cordyceps fumosorosea*, strain ESALQ 1296). The columns and bars represent the mean and the standard error for each variable and time point. Bars with an asterisk (*) indicate a statistically significant difference (P < 0.05) according to t-Student test or Mann–Whitney U test between untreated vs. treated psyllids in the same time point (*ns* non-significant).

The only difference observed for the sequential variables was in the 'Number of probes for the 1st E1': ACP treated with *C. fumosorosea* performed fewer probes before the first phloem salivation (E1) than untreated insects (U = 152,000; df = 1; P = 0.03) (Fig. 3).

### 15 h after spraying

The effects of *C. fumosorosea* on the stylet activities of the ACP were more apparent when EPG recordings were started 15 h after insect spraying (Supplementary Table S2) However, the differences were limited to non-phloem parameters such as the number (NWEI) of non-probing (t = 3.859; df = 1; P < 0.01) and intercellular stylet pathway (waveform C) (t = 3.849; df = 1; P < 0.01) activities. Treated ACP performed these activities less often than untreated insects, without affecting the duration (WDI) of the activities (Fig. 1).

Treated ACP spent more time (23.8 ± 6.9 min) to perform the ‘1st probe from start of EPG’ than untreated insects (8.4 ± 5.0 min) (t = − 3.328; df = 1; P < 0.01), and as well as at time point 0-h, treated ACP performed fewer probes to the 1st E1 than untreated insects (U = 159.500; df = 1; P < 0.03) (Fig. 3).

**Figure 4:**
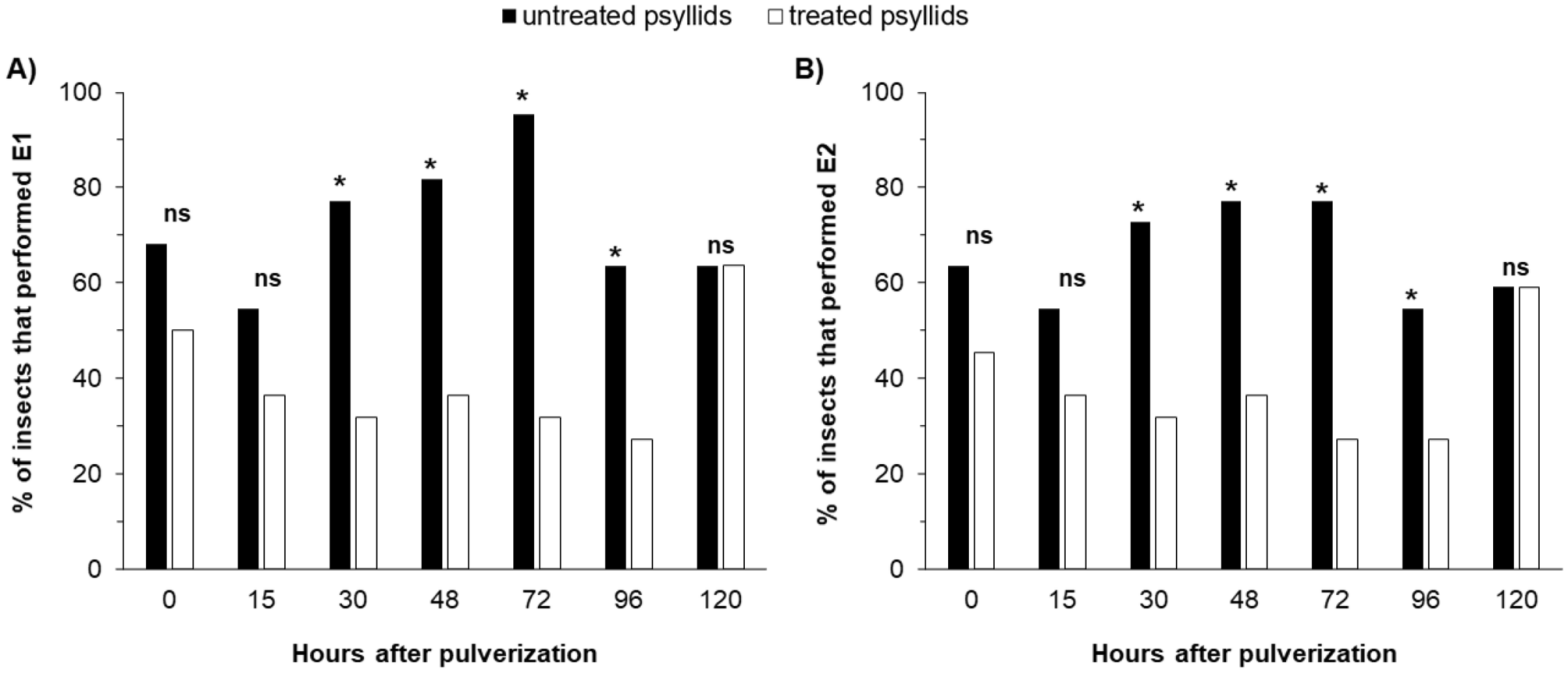
Proportion (PPW) of Asian-citrus-psyllids *Diaphorina citri* that generated salivation into phloem sieve elements (waveform E1) (**A**) and passive phloem-sap ingestion (waveform E2) (**B**) on ‘Rangpur lime’ seedlings at different time points after microbiological insecticide pulverization (*Cordyceps fumosorosea*, strain ESALQ 1296). *Bars with an asterisk* (*) indicate a statistically significant difference (P < 0.05) according to chi-square (x^2^) test with Bonferroni correction for specific pairwise comparisons.

No differences were observed between treatments in the PPW of E1 (x^2^ = 1.467; P = 0.36) and E2 (x^2^ = 1.467; P = 0.36) (Fig. 4).

### 30 h after spraying

The effects of the treatment with *C. fumosorosea* started to become more apparent and reached phloem parameters when EPG recordings were started 30 h after the insect was sprayed (Supplementary Table S3).

The proportion (PPW) of treated ACP that performed phloem-feeding activities was significantly lower compared to untreated insects, for both E1 (x^2^ = 9.167; P < 0.01) and E2 (x^2^ = 7.379; P = 0.01) (Fig. 4). Treated ACP that were able to reach the phloem vessels salivated (waveform E1) (NWEI: U = 157,000; df = 1; P = 0.03) and ingested (waveform E2) (NWEI: U = 162.500; df = 1; P = 0.04) phloem sap less often than untreated insects (Fig. 2). However, only the ingestion time was affected, and treated ACP ingested phloem sap for less than one hour, in contrast to untreated insects, which ingested phloem content for 2.5 h on average (WDI: U = 126.000; df = 1; P < 0.01) (Fig. 2). Thus, the fungus shortened the phloem phase (E = E1 + E2) (treated ACP: 48.8 ± 18.46 min; untreated ACP: 156.52 ± 27.93 min) (U = 113,500; df = 1; P = 0.02).

Unlike the earlier periods, 30 h after spraying, treated ACP took longer, from the beginning of the EPG recording, to perform the first activity in the phloem than the untreated ACP (U = 146,500; df = 1; P = 0.02) (Fig. 3).

### 48 h after spraying

Forty-eight hours after ACP spraying, both non-phloem and phloem-feeding activities were significantly affected by *C. fumosorosea* infection.

Treated ACP performed non-probing periods (waveform np) more often (NWEI: t = − 3.984; df = 1; P < 0.01) and the total duration of these periods was significantly longer (WDI: t = − 3.404; df = 1; P < 0.01) than in untreated ACP. Also, intercellular stylet pathway activities (waveform C) were performed more frequently by treated ACP than by untreated ones (NWEI: t = − 3.527; df = 1; P < 0.01), although without affecting the duration of this parameter (WDI: U = 163.000; df = 1; P = 0.06) (Fig. 1).

The proportion (PPW) of treated ACP that performed the phloem-feeding phase was significantly lower than in untreated ACP, for phloem salivation (waveform E1) (x^2^ = 9.402; P < 0.01) and phloem ingestion (waveform E2) (x^2^ = 7.503; P = 0.01) (Fig. 4). Treated ACP performed fewer phloem-salivation (waveform E1) (NWEI: U = 130.000; df = 1; P = 0.01) and phloem-ingestion (waveform E2) (NWEI: U = 130.500; df = 1; P < 0.01) activities than untreated psyllids (Fig. 2). The same tendency was observed for the total duration of the phloem salivation period (WDI: U = 122.000; df = 1; P < 0.01) and phloem ingestion period (WDI: U = 103.000; df = 1; P < 0.01), which were significantly shorter in treated than untreated psyllids (Fig. 2).

Differences were observed in all sequential parameters evaluated. The 'Time to 1st probe from starts of EPG' was shorter in treated ACP (19.19 ± 6.33 min) than untreated psyllids (40.29 ± 12.19 min) (t = 2.152; df = 1; P = 0.04). The 'Number of probes to the 1st E1' was high for treated than untreated ACP (t = − 4.397; df = 1; P < 0.01). The same tendency was observed for 'Time from start of EPG to 1st E' (U = 91.000; df = 1; P < 0.01) (Fig. 3; Supplementary Table S4).

### 72 h after spraying

As in the 48-h time point, the probing behavior of ACP sprayed 72 h before the beginning of EPG recording was affected, both in non-phloem (Fig. 1) and phloem activities (Fig. 2; Supplementary Table S5).

Treated ACP performed non-probing periods (waveform np) more often (NWEI: t = − 5.364; df = 1; P < 0.01); the total duration of these periods was three times longer (WDI: U = 73.000; df = 1; P < 0.01) (Figure) than in untreated psyllids. The number of stylet pathways (waveform C) was also higher for treated than for untreated ACP (NWEI: t = − 3.711; df = 1; P < 0.01), however without influencing the duration of this parameter (WDI: U = 73.000; df = 1; P = 0.09) (Fig. 1).

The proportion (PPW) of treated ACP that performed phloem activities was significantly lower compared to untreated ACP, for E1 (x^2^ = 19,250; P < 0.01) and E2 (x^2^ = 11.023; P < 0.01) (Fig. 4). Also, treated ACP performed fewer E1 (NWEI: U = 101.500; df = 1; P = 0.01) and E2 (NWEI: U = 100.500; df = 1; P < 0.01), with shorter durations ((WDI-E1: U = 91.500; df = 1; P < 0.01); (WDI-E2: U = 84.000; df = 1; P < 0.01) than untreated psyllids (Fig. 2). Furthermore, treated ACP performed three times more probes before the first E1 than untreated psyllids (t = − 5.481; df = 1; P < 0.01), as well as taking twice as long from the start of the EPG to the first contact with the phloem vessels than untreated insects did (U = 61.500; df = 1; P < 0.01) (Fig. 3).

### 96 h after spraying

Four days (96 h) after the insects were treated, only the non-probing duration was affected regarding non-phloem parameters: treated ACP remained three times longer with their stylets outside the plant tissue than did untreated psyllids (WDI: U = 59.000; df = 1; P < 0.01) (Fig. 1; Supplementary Table S6).

The proportion (PPW) of treated ACP that performed phloem salivation (E1 waveform) was significantly lower than in untreated insects (x^2^ = 5.867; P = 0.01) (Fig. 4). Also, treated insects performed fewer E1 (NWEI: U = 134,000; df = 1; P < 0.01) and E2 (NWEI: U = 159,500; df = 1; P = 0.03), and the duration of these activities was also significantly shorter ((WDI-E1: U = 133.000; df = 1; P < 0.01); (WDI-E2: U = 162.000; df = 1; P = 0.03) (Fig. 2).

The sequential parameters differed only in the 'Time from start of EPG to 1st E', which was longer in the treated ACP (U = 142.00; df = 1; P = 0.01) (Fig. 3).

### 120 h after spraying

Few effects on probing behavior were observed 5 days (120 h) after insects were treated. The non-probing time of treated ACP was longer than in untreated insects (NWEI: t = 1.832; df = 1; P = 0.04) (Fig. 1). Treated ACP also took three times longer to perform the 1st probe from the start of EPG (treated ACP 47.43 ± 12.47 min; untreated ACP 14.22 ± 4.64 min) (t = − 3.766; df = 1; P < 0.01) (Supplementary Table S7).

No differences were observed in parameters associated with phloem (Fig. 2) or in the PPW (Fig. 4) (P > 0.05).

Twenty-four hours after the beginning of the recordings, it was evaluated the number of living and dead insects connected to the EPG system in the control and in the treatment with *C. fumosorosea* at all time-points, in order to verify possible effects of product application. In the control treatment, the survival of the insects ranged from 90 to 100%, whereas in the treatment where the insects were sprayed with *C. fumosorosea* ranged from 63 to 95.4% (Table 1).

**Table 1:** Number and percentage of living and dead *Diaphorina citri* after 24 h of the beginning of EPG recordings in each treatments and timepoints.

Timepoint	Treatment			
	Untreated psyllids (n = 22)		Treated psyllids (n = 22)	
	Living psyllids (n)	Dead psyllids (n)	Living psyllids (n)	Dead psyllids (n)
0 h	20 (90.9%)	2 (9.1%)	14 (63.6%)	8 (36.4%)
15 h	21 (95.4%)	1 (4.5%)	18 (81.8%)	4 (18.2%)
30 h	21 (95.4%)	1 (4.5%)	16 (72.7%)	6 (27.3%)
48 h	21 (95.4%)	1 (4.5%)	17 (77.3%)	5 (22.7%)
72 h	22 (100%)	0 (0%)	19 (86.4%)	3 (13.6%)
96 h	22 (100%)	0 (0%)	16 (72.7%)	6 (27.3%)
120 h	21 (95.4%)	1 (4.5%)	21 (95.4%)	1 (4.5%)

## Discussion

The present study has shown firsthand the effects of *C. fumosorosea* on the probing behavior of *D. citri* using the EPG technique. The effects were more pronounced between 30 and 96 h after the insects were sprayed, with significant disruption of the stylet activities mainly in the phloem-vessels, directly associated with the transmission of HLB.

The use of entomopathogenic microorganisms such as fungi and bacteria has been suggested as an alternative to conventional control of agricultural pests, which is based on intensive applications of chemical insecticides, leading to several undesired secondary effects, including the selection of resistant populations to the available chemicals. In recent years there has been a significant increase in studies demonstrating that entomopathogens, especially fungi, play important roles in the ecosystem, as endophytes in plants^34^, growth promoters^45,46^, and phytopathogen antagonists^47,48^.

Much of the research has reported that members of the fungal order Hypocreales such as *Beauveria bassiana* (Hypocreales: Cordycipitaceae) (Bals. -Criv) Vuill. and *C. fumosorosea* act as important biological-control agents for different species of hemipterans, such as psyllids^24,25,27^, aphids^34,49^, and whiteflies^26,50^. The effects of these agents on insect behavior are still poorly understood, especially regarding insect vectors of plant diseases.

In general, entomopathogenic microorganisms require time to effectively infect their hosts and kill them, even though the capacity of entomopathogenic fungi to produce many types of secondary metabolites/mycotoxins during the initial phase of colonization of the insect body is well known^51–53^. The insects may continue to feed, causing damage due to probing behavior and transmission of phytopathogens^54^. Because of the longer time needed to kill the target insect, many producers opt for traditional insecticides, as they are apparently more “aggressive and efficient” (in the eyes of the producer) in terms of how quickly they kill the pest.

Most available insecticides (chemical and microbiological) can cause high mortality in the insect vector population^10,21,27,31,55^, however, many insecticides cannot affect the probing behavior soon enough to prevent stylet activities associated with transmission of phytopathogens to new host plants. Therefore, for successful control of insect vectors, it is necessary to understand the insect´s characteristics and mechanisms of action of insecticides on its mortality, but also the modes of transmission of plant pathogens and stylet activities associated with transmission^10,31,32,34,56^.

In general, research on insect vectors and entomopathogenic fungi is mainly focused on evaluating their effects on mortality and indirect quantification of feeding through honeydew excretion^21^, or on reducing the symptoms of phytopathogens on the plants^54^. From this information, it is not possible to determine if there really was a change in the stylet activities of the insects in these plants.

The effects of an entomopathogenic fungus on the stylet activities of an insect vector using the EPG technique were evaluated by Gonzáles-Mas et al.^34^. They evaluated the probing behavior of *Aphis gossypii* Glover (Hemiptera: Aphididae) and the transmission of the persistent-circulative *Cucurbit aphid-borne yellow virus persistent virus* (CABYV) (*Polerovirus*) and the non-persistent *Cucumber mosaic virus* (CMV) (*Cucumovirus*) in melon plants colonized endophytically by *B. bassiana*. They showed that endophytic *B*. *bassiana* can be used in integrated pest management programs to reduce virus transmission by *A. gossypii,* since transmission of both viruses was lower when receptor plants had been treated with a suspension of *B. bassiana* conidia.

Chen et al.^33^ observed changes in the probing behavior of the green peach aphid *Myzus persicae* (Sulzer) (Hemiptera: Aphididae) when infected with the entomopathogenic fungus *Pandora neoaphidis* (Remaud. & Hennebert) Humber (Entomophthoromycotina: Entomophthorales). The authors found that the number and total duration of brief intracellular probes (pd) were greater for infected than uninfected aphids, which suggests that *P. neoaphidis* infection may increase the acquisition of a non-persistent virus during such intracellular probes. On the other hand, if infected aphids spent less time ingesting phloem sieve (WDI E2), this can reduce the spread of persistent viruses, which are transmitted during phloem phase.

For transmission of HLB-associated bacteria, the longer the acquisition access period (AAP)^11^ and inoculation access period (IAP), the greater the transmission efficiency, although the insects can acquire the bacteria within shorter periods^57^. However, between pathogen acquisition from an infected plant and inoculation into a healthy plant, there is a period required for the insect to be able of inoculating the pathogen, known as the latency period^58^. The mean latency period for '*Candidatus* Liberibacter asiaticus' in adults of *D. citri* was estimated by Canale et al.^59^ as 17.8 days and the persistence of transmission by *D. citri* is approximately 5 weeks, which means that the insects can inoculate the bacterium into new plants for almost their entire life. Thus, there is a considerable period for entomopathogens to act before HLB transmission occurs.

Avery et al.^21^ observed that *D. citri* infected with *C. fumosorosea* produced significantly fewer honeydew droplets per day than the control psyllids, suggesting that they ate less when infected with the entomopathogenic fungus.

We observed that the stylet activities were considerably altered as soon as 30 h and the changes persisted until just over 96 h after spraying. At times shorter than 30 h and longer than 96 h, although the insects were "infected” with the fungus (as shown by the sporulation), the fungus did not disrupt the psyllids’ probing behavior regarding the phloem-feeding activities.

We hypothesize that before 30 h, the fungus did not have time to produce enough mycotoxins to disrupt the probing behaviors, and after 96 h of infection, although infected with *C. fumosorosea*, we assume that the psyllids that survive these days, may have been more resistant or had a defense system that prevented changes in their stylet activities, since, to infect the insect, the entomopathogenic fungi needs to display several steps, as adhesion of the spores, enzyme production, germination, hemocoel penetration, and replication. Therefore, the fungus must time to overstep the insect´s defense mechanisms to complete the infection process^60–62^.

Lei et al.^62^ evaluating the mode of action of *C. fumosorosea* on the diamondback moth larvae, *Plutella xylostella* (L.) (Lepdoptera: Plutellidae), observed that the first signs of germination started 4 h following inoculation and penetrate the epidermis and began to enter the hemocoel 24 h after. Thirty-six hours post-inoculation, the hyphal bodies colonized the insect body cavity, and 48 h post-inoculation the hyphae reproduced massively. This study shows that the entomopathogen needs time to effectively infect the host and alter its behavior.

The results from the use of the EPG technique indicated that the commercial product containing the fungus *C. fumosorosea* as the active ingredient disrupts the probing and stylet activities of *D. citri*. This product has the potential to reduce the psyllid´s capacity for HLB transmission and can be used as part of integrated pest and citrus disease management, being compatible with other control strategies. More detailed studies are needed to better understand these relationships among fungus colonization, secondary metabolites, and probing behavior.

## Supplementary Information

Supplementary Information.

## Data Availability

All data generated or analysed during this study are included in this published article and its supplementary material files.
