# Supplementary material for: Entomopathogenic fungus disrupts the phloem-probing behavior of Diaphorina citri and may be an important biological control tool in citrus
Source: Sci Rep. 2022 May 13;12:7959. doi: 10.1038/s41598-022-11789-2 (PMC9106691; doi:10.1038/s41598-022-11789-2)
Supplement: Supplementary file 1 — Supplementary Information. [file 41598_2022_11789_MOESM1_ESM.pdf]

Entomopathogenic fungus disrupts the phloem-probing behavior of *Diaphorina citri* and may be an important biological control tool in citrus

Nathalie Maluta[1] *, Thiago Castro[2], João Roberto Spotti Lopes[1]

[1]Department of Entomology and Acarology, Luiz de Queiroz College of Agriculture, University of São Paulo, C.P. 9, 13418-900, Piracicaba, SP, Brazil; nathaliepradomaluta@gmail.com; jrslopes@usp.br

[2] Koppert Biological Systems, Rodovia Margarida da Graça Martins s/n - Km 17,5, 13400-970, Piracicaba, SP, Brazil tcastro@koppert.com.br

*****Correspondence: nathaliepradomaluta@gmail.com ; Tel: +55 19 3429-4199 r.214 (N.M)

Table S1. Mean (± SEM) of non-sequential and sequential EPG variables for 8-h recordings of the probing behavior of *Diaphorina citri* on 'Rangpur lime' seedlings immediately after sprayed with *Cordyceps fumosorosea*

EPG non-sequential variables	Control 0h n= 22	Challenger 0h n= 22	*P*-value[b]	U or t* value
**NPI[a]**	19.91 ± 2.75	19.41 ± 3.11	0.76	0.303*
**NWEI[a]**				
np	20.55 ± 2.73	19.95 ± 3.13	0.67	0.422*
C	22.32 ± 2.74	20.59 ± 2.99	0.57	0.571*
G	0.27 ± 0.10	0.14 ± 0.07	0.27	209.000
D	2.14 ± 0.63	1.55 ± 0.58	0.26	196.000
E1	2.23 ± 0.64	1.59 ± 0.62	0.23	192.500
E2	0.91 ± 0.19	1.09 ± 0.47	0.35	205.000
E2s	0.68 ± 0.12	0.73 ± 0.29	0.32	204.000
**WDI (min)[a]**				
Probe	243.42 ± 22.15	241.09 ± 25.83	0.95	0.069
np	236.58 ± 22.15	238.91 ± 25.83	0.95	-0.069*
C	172.34 ± 19.81	241.08 ± 41.02	0.31	-1.031*
G	20.84 ± 11.60	5.85 ± 3.95	0.26	208.000
D	1.01 ± 0.27	0.53 ± 0.17	0.13	179.500
E1	2.05 ± 0.45	2.49 ± 1.51	0.18	187.500
E2	48.19 ± 18.02	31.50 ± 15.69	0.21	191.000
Total duration of E	50.24 ± 18.20	33.99 ± 17.18	0.19	188.500
**EPG Sequential variable**				
Time to 1st probe from start of EPG	14.17 ± 4.44	10.15 ± 2.49	0.92	238.000
Number of probes to the 1st E1	6.82 ± 1.41	2.77 ± 0.74	<u>0.03</u>	152.000
Time from start of EPG to 1st E	245.39 ± 36.36	275.96 ± 44.72	0.67	224.500

[a] **NPI:** number of probe per insect; **NWEI,** number of waveform events per insect; **WDI,** total waveform duration (min) per insect; [b] Statistical comparisons between treatments for each parameter were made by Student´s t-test (*) for Gaussian distribution variables or non-parametric Mann-Whitney U-test for non-Gaussian distribution variables. Underline-type indicates significant differences ($P < 0.05$) (Backus et al., 2007).

Table S2. Mean (± SEM) of non-sequential and sequential EPG variables for 8-h recordings of the probing behavior of *Diaphorina citri* on ‘Rangpur lime’ seedlings that were sprayed with *Cordyceps fumosorosea* 15 h earlier

EPG Variable	Controle 15h n= 22	Challenger 15h n= 22	*P*-value[b]	U or t* value
**NPI**[a]	19.77 ± 2.24	10.23 ± 1.83	<0.01	4.064*
**NWEI**[a]				
np	20.41 ± 2.20	10.68 ± 1.86	<0.01	3.859*
C	20.73 ± 2.21	11.59 ± 1.68	<0.01	3.849*
G	0.18 ± 0.08	0.77 ± 0.17	< 0.01	137.000
D	0.77 ± 0.19	0.73 ± 0.23	0.55	219.000
E1	0.77 ± 0.19	0.73 ± 0.23	0.55	219.000
E2	0.64 ± 0.14	0.50 ± 0.16	0.37	208.000
E2s	0.59 ± 0.13	0.41 ± 0.13	0.26	200.000
**WDI (min)**[a]				
Probe	265.20 ± 21.37	296.87 ± 25.55	0.18	185.000
np	214.80 ± 21.37	183.13 ± 25.55	0.18	185.000
C	231.17 ± 19.83	187.21 ± 21.30	0.14	1.511*
G	2.86 ± 1.59	52.63 ± 15.13	< 0.01	122.000
D	0.40 ± 0.10	0.63 ± 0.22	0.84	234.000
E1	1.05 ± 0.25	1.47 ± 0.52	0.64	224.000
E2	29.72 ± 19.68	54.93 ± 19.48	0.84	234.000
Total duration of E	30.77 ± 9.79	56.39 ± 19.91	0.80	234.000
**EPG Sequential variable**				
Time to 1st probe from start of EPG	8.41 ± 5.00	23.84 ± 6.90	< 0.01	-3.328*
Number of probes to the 1st E1	7.45 ± 1.66	1.82 ±0.78	0.03	159.500
Time from start of EPG to 1st E	334.81 ± 30.09	389.69 ± 28.59	0.19	191.000

[a] **NPI:** number of probe per insect; **NWEI,** number of waveform events per insect; **WDI,** total waveform duration (min) per insect; [b] Statistical comparisons between treatments for each parameter were made by Student´s t-test (*) for Gaussian distribution variables or non-parametric Mann-Whitney U-test for non-Gaussian distribution variables. Underline-type indicates significant differences ($P < 0.05$) (Backus et al., 2007).

Table S3. Mean (± SEM) of non-sequential and sequential EPG variables for 8-h recordings of the probing behavior of *Diaphorina citri* on 'Rangpur lime' seedlings that were sprayed with *Cordyceps fumosorosea* 30 h earlier

**EPG Variable**	**Controle n= 22**	**Challenger n= 22**	***P*-value[b]**	**U or t* value**
**NPI[a]**	7.09 ± 1.26	5.36 ± 0.93	0.28	1.095*
**NWEI[a]**				
np	7.41 ± 1.28	5.68 ± 0.99	0.31	1.021*
C	8.18 ± 1.26	6.68 ± 0.83	0.53	0.625
G	0.27 ± 0.13	0.64 ± 0.17	0.05	171.000
D	1.23 ± 0.21	0.82 ± 0.31	<u>0.04</u>	159.500
E1	1.32 ± 0.24	0.82 ± 0.31	<u>0.03</u>	157.000
E2	1.00 ± 0.15	0.64 ± 0.24	<u>0.04</u>	162.500
E2s	0.91 ± 0.15	0.50 ± 0.18	<u>0.04</u>	164.500
**WDI (min)[a]**				
Probe	298.92 ± 21.12	290.03 ± 30.25	0.81	0.241*
np	181.08 ± 21.12	189.97 ± 30.25	0.81	-0.241*
C	134.09 ± 22.50	196.10 ± 28.85	0.13	-1.546*
G	7.47 ± 3.82	44.52 ± 17.71	<u>0.02</u>	157.000
D	0.83 ± 0.19	0.61 ± 0.21	0.07	168.500
E1	2.10 ± 0.76	1.59 ± 0.68	0.06	163.500
E2	154.41 ± 27.81	47.20 ± 17.85	<u>< 0.01</u>	126.000
Total duration of E	156.52 ± 27.93	48.80 ± 18.46	<u>< 0.01</u>	113.500
**EPG Sequential variable**				
Time to 1st probe from start of EPG	34.93 ± 9.42	32.85 ± 7.74	0.62	-0.499*
Number of probes to the 1st E1	6.05 ± 1.22	5.18 ± 0.96	0.48	0.713*
Time from start of EPG to 1st E	266.55 ± 30.80	383.47 ± 35.39	<u>0.02</u>	146.500

[a]**NPI:** number of probes per insect; **NWEI,** number of waveform events per insect; **WDI,** total waveform duration (min) per insect; [b] Statistical comparisons between treatments for each parameter were made by Student´s t-test (*) for Gaussian distribution variables or non-parametric Mann-Whitney U-test for non-Gaussian distribution variables. Underline-type indicates significant differences ($P < 0.05$) (Backus et al., 2007).

Table S4. Mean (± SEM) of non-sequential and sequential EPG variables for 8-h recordings of the probing behavior of *Diaphorina citri* on 'Rangpur lime' seedlings that were sprayed with *Cordyceps fumosorosea* 48 h earlier

EPG Variable	Controle 48h n= 22	Challenger 48h n= 22	*P*-value[b]	U or t* value
**NPI[a]**	7.45 ± 1.70	19.27 ± 3.67	<0.01	-3.861*
**NWEI[a]**				
np	7.55 ± 1.74	19.77 ± 3.69	<0.01	-3.984*
C	9.45 ± 1.68	20.59 ± 3.54	<0.01	-3.527*
G	0.41 ± 0.14	0.64 ± 0.14	0.18	192.000
D	2.18 ± 0.40	0.82 ± 0.27	< 0.01	127.500
E1	2.14 ± 0.40	0.82 ± 0.27	0.01	130.000
E2	1.09 ± 0.19	0.41 ± 0.13	< 0.01	130.500
E2s	1.00 ± 0.19	0.41 ± 0.13	0.012	145.000
**WDI (min)[a]**				
Probe	357.30 ± 16.27	264.04 ± 22.04	<0.01	3.404
np	122.70 ± 16.27	215.96 ± 22.04	<0.01	-3.404*
C	135.26 ± 24.84	189.63 ± 22.44	0.06	163.000
G	12.95 ± 4.21	25.47 ± 6.61	0.21	194.000
D	2.23 ± 0.79	0.74 ± 0.32	< 0.01	132.000
E1	2.13 ± 0.43	0.82 ± 0.29	< 0.01	122.000
E2	204.73 ± 33.67	47.37 ± 18.68	<0.01	103.000
Total duration of E	206.86 ± 33.83	48.19 ± 18.89	<0.01	96.000
**EPG Sequential variable**				
Time to 1st probe from start of EPG	40.29 ± 12.19	19.19 ± 6.33	0.04	2.152*
Number of probes to the 1st E1	6.59 ± 1.72	19.18 ± 3.68	<0.01	-4.397*
Time from start of EPG to 1st E	214.21 ± 33.89	404.86 ± 24.42	<0.01	91.000

[a] **NPI:** number of probe per insect; **NWEI,** number of waveform events per insect; **WDI,** total waveform duration (min) per insect; [b] Statistical comparisons between treatments for each parameter were made by Student´s t-test (*) for Gaussian distribution variables or non-parametric Mann-Whitney U-test for non-Gaussian distribution variables. Underline-type indicates significant differences ($P < 0.05$) (Backus et al., 2007).

Table S5. Mean (± SEM) of non-sequential and sequential EPG variables for 8-h recordings of the probing behavior of *Diaphorina citri* on 'Rangpur lime' seedlings that were sprayed with *Cordyceps fumosorosea* 72 h earlier

EPG Variable	Controle 15h n= 22	Challenger 15h n= 22	*P*-value[b]	U or t* value
**NPI**[a]	5.23 ± 0.83	18.09 ± 3.14	<0.01	-5.202
**NWEI**[a]				
np	5.36 ± 0.87	18.68 ± 3.17	<0.01	-5.364
C	7.86 ± 1.09	18.95 ± 3.15	0.01	-3.711
G	0.73 ± 0.15	0.27 ± 0.10	0.02	156.000
D	2.50 ± 0.58	0.77 ± 0.26	0.01	108.500
E1	2.59 ± 0.62	0.73 ± 0.26	0.01	101.000
E2	1.41 ± 0.24	0.32 ± 0.12	<0.01	100.500
E2s	1.14 ± 0.18	0.32 ± 0.12	0.01	108.000
**WDI (min)**[a]				
Probe	404.82 ± 13.18	250.36 ± 26.49	<0.01	73.000
np	75.18 ± 13.18	229.64 ± 26.49	<0.01	73.000
C	155.55 ± 27.84	205.15 ± 24.62	0.09	-1.718
G	22.21 ± 4.85	12.41 ± 5.25	0.08	175.000
D	1.82 ± 0.41	0.34 ± 0.12	<0.01	74.500
E1	5.03 ± 1.98	1.81 ± 1.13	<0.01	91.500
E2	220.21 ± 33.12	30.65 ± 16.39	<0.01	84.000
Total duration of E	225.24 ± 33.07	32.46 ± 4.65	<0.01	55.500
**EPG Sequential variable**				
Time to 1st probe from start of EPG	21.00 ± 7.50	14.58 ± 4.65	0.67	0.425
Number of probes to the 1st E1	4.14 ± 0.69	17.27 ± 3.22	<0.01	-5.481
Time from start of EPG to 1st E	176.05 ± 27.60	407.82 ± 28.35	<0.01	61.500

[a]**NPI:** number of probes per insect; **NWEI,** number of waveform events per insect; **WDI,** total waveform duration (min) per insect; [b] Statistical comparisons between treatments for each parameter were made by Student´s t-test (*) for Gaussian distribution variables or non-parametric Mann-Whitney U-test for non-Gaussian distribution variables. Underline-type indicates significant differences ($P < 0.05$) (Backus et al., 2007).

Table S6. Mean (± SEM) of non-sequential and sequential EPG variables for 8-h recordings of the probing behavior of *Diaphorina citri* on 'Rangpur lime' seedlings that were sprayed with *Cordyceps fumosorosea* 96 h earlier

EPG Variable	Controle 15h n= 22	Challenger 15h n= 22	*P*-value[b]	U or t* value
**NPI**[a]	9.41 ± 1.41	12.23 ± 1.71	0.258	-1.147*
**NWEI**[a]				
np	9.50 ± 1.44	12. 82 ± 1.76	0.17	-1.410*
C	11.05 ± 1.35	12.59 ± 1.79	0.67	-0.425
G	0.36 ± 0.12	0.14 ± 0.07	0.14	196.500
D	1.77 ± 0.41	0.45 ± 0.19	<0.01	137.000
E1	1.82 ± 0.43	0.41 ± 0.17	< 0.01	134.000
E2	1.14 ± 0.30	0.32 ± 0.12	0.03	159.500
E2s	0.82 ± 0.20	0.23 ± 0.09	0.03	161.000
**WDI (min)**[a]				
Probe	394.02 ± 14.63	242.82 ± 22.70	<0.01	59.000
np	85.98 ± 14.63	237.18 ± 22.70	<0.01	59.000
C	269.48 ± 26.99	213.92 ± 24.89	0.17	184.000
G	22.64 ± 13.23	4.34 ± 2.50	0.15	197.500
D	1.45 ± 0.40	0.50 ± 0.28	<0.01	138.000
E1	2.63 ± 0.68	0.49 ± 0.24	<0.01	133.000
E2	98.55 ± 26.94	32.03 ± 15.16	0.03	162.000
Total duration of E	101.18 ± 27.29	32.52 ± 15.22	0.01	146.000
**EPG Sequential variable**				
Time to 1st probe from start of EPG	12.42 ± 3.04	28.75 ± 11.25	0.35	-0.952
Number of probes to the 1st E1	9.00 ± 1.42	12.23 ± 1.71	0.17	-1.396*
Time from start of EPG to 1st E	339.49 ± 34.62	435.84 ± 18.00	0.01	142.000

[a] **NPI:** number of probe per insect; **NWEI,** number of waveform events per insect; **WDI,** total waveform duration (min) per insect; [b] Statistical comparisons between treatments for each parameter were made by Student´s t-test (*) for Gaussian distribution variables or non-parametric Mann-Whitney U-test for non-Gaussian distribution variables. Underline-type indicates significant differences ($P < 0.05$) (Backus et al., 2007).

Table S7. Mean (± SEM) of non-sequential and sequential EPG variables for 8-h recordings of the probing behavior of *Diaphorina citri* on 'Rangpur lime' seedlings that were sprayed with *Cordyceps fumosorosea* 120-h earlier

**EPG Variable**	**Controle 15h n= 22**	**Challenger 15h n= 22**	***P*-value[b]**	**U or t* value**
**NPI[a]**	10.05 ± 1.94	5.82 ± 0.86	0.12	1.594*
**NWEI[a]**				
np	10.32 ± 1.92	5.91 ± 0.87	0.04	1.832
C	11.68 ± 1.96	8.09 ± 0.89	0.29	1.061*
G	0.27 ± 0.12	0.77 ± 0.16	0.01	150.500
D	1.64 ± 0.38	2.05 ± 0.54	0.83	233.000
E1	1.50 ± 0.34	1.91 ± 0.47	0.72	227.500
E2	0.91 ± 0.22	0.95 ± 0.23	0.91	237.500
E2s	0.77 ± 0.19	0.77 ± 0.17	0.91	237.500
**WDI (min)[a]**				
Probe	331.07 ± 18.18	298.17 ± 21.87	0.25	1.157*
np	148.93 ± 18.18	181.83 ± 21.87	0.25	-1.157*
C	219.15 ± 26.20	206.78 ± 27.76	0.75	0.324*
G	9.04 ± 4.03	25.29 ± 5.26	0.01	149.500
D	1.35 ± 0.28	1.45 ± 0.43	0.70	226.000
E1	2.23 ± 0.51	2.96 ± 0.76	0.75	229.000
E2	99.30 ± 27.71	61.69 ± 18.52	0.53	216.500
Total duration of E	101.53 ± 27.88	64.65 ± 18.99	0.56	218.000
**EPG Sequential variable**				
Time to 1st probe from start of EPG	14.22 ± 4.64	47.43 ± 12.47	< 0.01	-3.766*
Number of probes to the 1st E1	8.91 ± 1.85	5.41 ± 0.91	0.14	1.506*
Time from start of EPG to 1st E	312.98 ± 34.93	342.57 ± 30.99	0.61	221.000

[a] **NPI:** number of probe per insect; **NWEI,** number of waveform events per insect; **WDI,** total waveform duration (min) per insect; [b] Statistical comparisons between treatments for each parameter were made by Student´s t-test (*) for Gaussian distribution variables or non-parametric Mann-Whitney U-test for non-Gaussian distribution variables. Underline-type indicates significant differences ($P < 0.05$) (Backus et al., 2007).
